# Organic Nanomaterials and Their Applications in the Treatment of Oral Diseases

**DOI:** 10.3390/molecules21020207

**Published:** 2016-02-09

**Authors:** Maria Justina Roxana Virlan, Daniela Miricescu, Radu Radulescu, Cristina M. Sabliov, Alexandra Totan, Bogdan Calenic, Maria Greabu

**Affiliations:** 1 Department of Biochemistry, Faculty of Dentistry, University of Medicine and Pharmacy Carol Davila, Blvd. EroiiSanitari, No. 8, RO-050474 Bucharest, Romania; mjr.virlan@gmail.com (M.J.R.V.); miricescudaniela@yahoo.com (D.M.); radu_radulescu24@yahoo.com (R.R.); alexandratotan@yahoo.com (A.T.); mariagreabu@yahoo.com (M.G.); 2 Agricultural and Biological Engineering Department, Louisiana State University and LSU Ag Center, 149 EB Doran Building, Baton Rouge, LA 70803, USA; CSabliov@agcenter.lsu.edu

**Keywords:** nanoparticles, organic, dentistry, polymers, regeneration

## Abstract

There is a growing interest in the development of organic nanomaterials for biomedical applications. An increasing number of studies focus on the uses of nanomaterials with organic structure for regeneration of bone, cartilage, skin or dental tissues. Solid evidence has been found for several advantages of using natural or synthetic organic nanostructures in a wide variety of dental fields, from implantology, endodontics, and periodontics, to regenerative dentistry and wound healing. Most of the research is concentrated on nanoforms of chitosan, silk fibroin, synthetic polymers or their combinations, but new nanocomposites are constantly being developed. The present work reviews in detail current research on organic nanoparticles and their potential applications in the dental field.

## 1. Introduction

In the recent years, engineered nanoparticles have raised substantial interest due to their possible medical applications in vaccination, diagnostic imaging procedures [1], cancer therapy [2] or sustained delivery of drugs [3]. Nanomaterials, *i.e.,* materials with components less than 100 nm in at least one dimension [4,5], include nanocontainers, nanofilms, nanomembranes, nanoscaffolds or composites that are a combination of these. Nanoparticles have all three external dimensions in the nanoscale and exhibit characteristics distinct from the corresponding bulk material. Biomedicine stands to profit from the use of nanocarriers. Some of the advantages of the nanostructures are: higher colloidal stability, improved dispersibility, and improved surface reactivity. The most important characteristic of nanoparticles continues to be their ability to control delivery of drugs such as small molecule drugs, proteins, and DNA [6,7].

**Figure 1:**
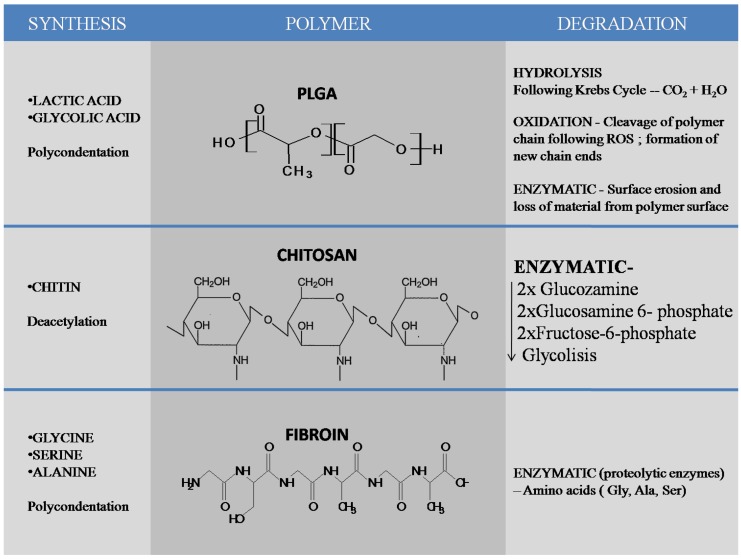
Synthesis and degradation of most important polymers used in formation of nanomaterials most commonly used in the dental field, PLGA, chitosan and silk fibroin

In dentistry, drug-loaded nano-pharmaceuticals have been extensively utilized over the past few years and are studied in almost all dental related fields [8]. A considerable amount of research has been conducted on metallic nanoparticles, but their safety is still under discussion [9,10,11]. Because biomedical nanoparticles should be nontoxic for cells (either bioinert or biodegradable), and because their use should not cause side effects in other tissues [12], multiple research groups have shifted their focus from metallic to organic nanoparticles, such as chitosan, silk fibroin or other biodegradable polymers, including poly(lactic-co-glycolic) acid (PLGA). PLGA is a copolymer synthesized from two different monomers—lactic and glycolic acids. PLGA can be obtained mainly by ring opening polymerization and polycondensation. Hydrolysis, oxidation and enzymatic degradation are the most important mechanisms of PLGA degradation. Chitosan is a linear polysaccharide composed of β-(1-4)-linked d-glucosamine (deacetylated unit) and *N*-acetyl-d-glucosamine(acetylated unit) obtained from chitin by partial deacetylation. Enzymatic degradation of chitosan leads to glucosamine which is phosphorylated to glucosamine 6-phosphate, deaminated and izomerized tofructose-6-phosphate which enters glycolysis. Silk fibroin (SF) is a natural polymer obtained by a variety of species including silkworms (the domesticated silkworm Bombyxmori) and spiders. Enzymatic degradation of the molecules results in amino-acids as end products without modifying the pH. Chitosan and silk fibroin are natural materials, while poly(lactic-co-glycolic) acid is a synthetic polymer that has been approved, due to its biocompatibility, by the U.S. Food and Drug Administration and European Medicine Agency [13] (Figure 1). The focus of this paper is to present the recent advances on organic nanomaterials made of natural compounds (chitosan, chitosan composites and silk fibroin) and synthetic materials (PLGA and composites thereof) and their use in dental nanomedicine.

## 2. Main Organic Nanomaterials Used in the Dental Field

### 2.1. Natural Organic Nano-Delivery Systems

Because of their increased biocompatibility, preference for natural products in biomaterial research is increasing. There is an international resurgence of interest in natural products for the development of novel drugs and therapies that could have medical applications [14], some of which are described below. Chitosan is obtained through partial deacetylation from chitin which is a positively charged polysaccahride [15] found in crustaceans [16,17]. It exhibits impressive characteristics for drug delivery applications: biocompatibility, low cost, mucus adhesion, and no immunogenicity or cytotoxicity [18,19,20,21,22]. Silk fibroin, another natural biomaterial, has recently been studied as a substrate for tissue engineered cartilage, bone, ligaments, nerves and also for drug delivery applications. Silk is a naturally occurring polymer material with a high resistance to deformation [23], while silk fibroin is a structural protein isolated from silk fibers separated from the cocoons of the silkworm *Bobymexmori* [24,25].

### 2.2. Chitosan Nanoparticles (CNPs)

In recent years, there has been an increasing interest in using chitosan and chitin for dental medicine applications. A number of studies focused on their controlled delivery properties as well as their ability to support regeneration of oral tissues with applications that span almost all major fields of dentistry: endodontics, periodontics, regenerative dentistry, invasive dentistry or even implantology (Table 1).

**Table 1:** Applications of chitosan nanoparticles in the oral field.

Chitosan Nanosystems	Application	Year	Study
Bone morphogenetic protein-2	Bone regeneration	2015	[26]
Bone morphogenetic protein 7	Bone regeneration	2015	[27]
Protein growth factors	Bone regeneration	2014	[28]
Dexamethasone	Dentin pulp regeneration	2015	[29]
Cetylpyridinium chloride and naf	Dental toothpastes	2015	[30]
Chlorhexidinedihydrochloride	Dental toothpastes	2015	[30]

Recent work shows that chitosan nanoparticles could be used in new bone formation therapies, leading the way for future applications in implantology, periodontology or dental surgery. Researchers developed bioactive scaffolds containing CNPs incorporated with protein growth factors for bone tissue regeneration [26,28,31], with promising *in vivo* results. **T**itanium implants coated with chitosan nanoparticles loaded with biologically active bone morphogenetic protein-2 (BMP-2) managed to induce ectopic bone growth on mice [26]. Recently, a poly(ε-caprolactone) nanofibrous implant functionalized with a chitosan nano-reservoir containing bone morphogenetic protein 7 implanted together with human mesenchymal stem cells resulted in new bone formation and calcification in mice calvarial defects [27]. And interestingly, decorating nanofibers of collagen with protein growth factor loaded chitosan nanocontainers accelerated the speed of bone regeneration *in vivo* [28]. Moreover, a composite hydrogel containing 2-*N*,6-*O*-sulfatedchitosan (26SCS) nanoparticles loaded with bone morphogenetic protein-2 was shown to have profound osteogenic activity, producing mature compact bone associated with new vascular in growth in ectopic bone [32]. This promising result might be due to the fact that 26SCS2-*N*,6-*O*-sulfated chitosan(26SCS) promotes the BMP-2 signaling pathway [33], suggesting that 26SCS could be used as the synergistic factor of BMP-2 for bone regeneration. Other data demonstrate that chitosan nanofibers can stimulate osteoblast proliferation and maturation via runt-related transcription factor 2-mediated regulation of osteoblast-associated osteopontin, osteocalcin, and alkaline phosphatase (ALP) gene expression [34]. Researchers observed that mouse osteoblasts grew much better on chitosan nanofiber scaffolds than on chitosan films. Moreover, nanofibers of phosphate functionalized derivatives ofchitosan-*N*-methylene phosphonic chitosan managed to accelerate bone healing by 300% compared to controls in rabbit tibial defects [35]. A recent study stated that cortical bone allografts coated with chitosan nanofibers could serve as periosteum mimics in bone grafting procedures [36]. Regenerative dentistry can also profit from the CNPs’ properties of temporal-controlled release of bioactive molecules [29,37] as they are successfully used in studies concerning dentin pulp regeneration. Bovine serum albumin loaded chitosan nanoparticles were proven to regulate the alkaline phosphatase activity (ALP) in stem cells from apical papilla, while chitosan nanoparticles incorporating dexamethasone have been able to stimulate the differentiation of human dental stem cells from apical papilla into odontoblast-like cells [37].

Minimally invasive dentistry is another field where chitosan nanoparticles could be used to remineralize early enamel caries [38]. A biomimetic remineralizing solution containing nanocomplexes of phosphorylated chitosan and amorphous calcium phosphate led to results similar to fluoride on enamel remineralization, but at a higher rate [38]. Its application could also reduce the risks associated with fluoride treatment, such as allergies or fluorosis [39].

Chitosan nanoparticles exhibit antibacterial proprieties mainly because of their polycationic charge, with higher reactivity in nanoform than in bulk [40,41,42,43]. This quality has broadened the research of chitosan nanoparticles in dental endodontics, as they can be successfully used in root canal treatments. CNPs possess an inherent antibacterial activity effective against *Enterococcus faecalis* [44], a property that is not diminished in the presence of dentin or lipopolysaccharides [41]. Moreover, incorporation of chitosan nanoparticles into root canal sealers has the potential benefits of inhibiting microbial penetration and reduced biofilm formation at the dentin-root filling interface [45,46]. Consequently, several endondotic sealers have been developed by incorporating chitosan nanoparticles into zinc-oxide-eugenol [47], epoxy resin or calcium silicate-based sealers [45], with each presenting enhanced antibacterial qualities. However, in a recent study, resin-based dental sealants modified with nylon-6 and chitosan nanofibers were prepared in an attempt to provide an antibacterial effect, but none of the chitosan-containing sealants displayed antimicrobial proprieties [48]. Also, chitosan nanoparticles showed a higher reduction of *Enterococcusfaecalis* biofilms as compared to calcium hydroxide, but bacteria still survived even after a 24 h treatment with 20 mg/mL chitosan nanoparticles [43]. It should also be noted that some researchers are not sure whether the inhibition of bacterial adherence by the chitosan nanoparticles is caused by killing the bacteria in their vicinity or by the nanoparticles’ direct effect on the bacteria-substrate interaction [47].

In the future, chitosan nanoparticles could be integrated into toothpastes or even used in dental prophylactic therapies aimed at reducing bacterial biofilms in the oral cavity. CNPs have already been developed that are loaded with toothpaste active compounds [30,49]. The *in vitro* toxicity of the chitosan nanoparticles on human gingival fibroblasts was considered moderate after 24 h exposure [30]. In addition, one study found that nanoparticle complexes prepared from low molecular weight chitosan showed a high antimicrobial effect on *Streptococcus mutans* biofilms [50]. CNPs were active at a neutralpH and resulted in damage to more than 95% of the *S.mutans* cells [50]. Because *Streptococcus mutans* is one of the most intensively studied cariogenic microorganisms associated with caries progression in humans [51,52], killing this bacterium could be an effective form of preventive dentistry.

Chitosan nanofibers, nanopowders and nanoparticles also showed promising results in other applications connected to the dental field, such as nerve regeneration medicine [53] or healing skin [54,55] and oral mucosa [56]. A chitosan-based nanofibrous material was tested as a wound dressing material for IIIa and IIIb degree burns and managed to protect the site from infection while also supporting skin regeneration [55]. Recently, a scaffold with a surface layer consisting of chitosan nanofibers was developed as a potential skin substitute [54].

The possible applications of CNPs in anticancer treatment should also be noted. Chitosan nanoparticles have been successfully incorporated into systems involving active targeting, controlled drug delivery, or imaging of cancer cells [57,58,59,60,61]. Methotrexate-charged chitosan nanoparticles have been formulated, and their utilization induced a greater increase of the apoptosis of tumour cells than treatment with the free drug [61]. Also, methotrexate CNPs exhibited relative selectivity for cancer cells, as the cytotoxic effects were significantly lower in the non-tumor control cells HaCaT (human keratinocytes immortalized) [61]. As methotrexate is a major chemotherapeutic used in the treatment of the head and neck cancers [62], CNPs loaded with anticancer drugs could be used in the future for oral cancer treatment. Interestingly, nanoparticles with surfaces decorated with chitosan that can specifically target theCD44receptors in cancer stem-like cells have recently been fabricated [63]. The CD44 receptor protein is found to be overexpressed by many tumors and is identified as one of the most common cancer stem cell surface markers in tumors including head and neck cancer [64,65]. Considering the fact that cancer stem cells in oral squamous carcinoma show high expression of CD44 [66], some might consider in the future the hypothesis that CNPs could be used for selective oral cancer stem cell targeting. However this hypothesis needs further testing in order to clearly show that CNP might be selective towards CD44 positive cells.

Chitosan nanoparticles applications in regenerative medicine as well as in regenerative dentistry is clearly an intensively studied field. Taking into account the high number of *in vitro*, but also *in vivo* presented studies, a next logical step should be the development of novel CNPs uses in dentistry as well as the beginning of future clinical trials.

### 2.3. Composite Chitosan Nanoparticles

Because chitosan nanoparticles exhibit several disadvantages, such as poor mechanical and processing properties or insolubility in common organic solvents [67], researchers have attempted to combine them with an impressive number of polymeric or inorganic or other organic substances. Table 2 briefly presents the main composite chitosan nanostructures that have been fabricated in recent years and could have potential uses in dentistry. Composite materials blend the advantages of chitosan with the added substance, and their nanosized presentation has shown desirable characteristics in biomedical applications. However, their uses are limited to bone regeneration and wound healing acceleration, with very few focusing on antibacterial treatments.

Because natural polymers exhibit similarities to the extracellular matrix, special attention is given to chitosan-based materials for bone tissue engineering application. Chitosan-gold nanoparticles managed to enhance osseointegration of dental implants [68,69,70] incorporated with the transcription factor c-myb or with the anti-inflammatory molecule peroxisome proliferator activated receptor gamma (PPAR). Interestingly, chitosan-gold nanoparticles conjugated with PPAR were used to modify dental implants *in vitro*, but also *in vivo* in rat mandible. The modified implants led to newly-formed bone with enhanced mineral density and reduced inflammation, and as such, may have future uses as a dental prosthetic material in patients suffering from inflammatory diseases like arthritis, diabetes and osteoporosis. Since the anti-inflammatory effect of peroxisome proliferator activated receptor gamma has been demonstrated in periodontitis [71,72], in human dental pulp cells [73,74] and in mouse osteoblast precursor cells [75], chitosan-gold nanoparticles incorporated with PPAR gamma could be tested in the future also for periodontal or pulpal regeneration.

Other composite chitosan nanoparticles have also been developed for bone tissue engineering. Recent studies focused on bone regeneration that could be supported by poly(ε-caprolactone) (PCL) nanofiber scaffolds containing chitosan nanoparticles or by chitosan-PCL nanofibers [76,77]. Scaffolds fabricated from poly(ε-caprolactone) (PCL) nanofibers and chitosan-PCL copolymers showed that the polymer is biocompatible with bone cells such as MG63 cells [76,78]. Moreover, nanofiber matrices of polycaprolactone-chitosan functionalized with collagen I led to higher alkaline phosphatase (ALP) activity and mineralization of rat bone marrow-derived stromal cells [77]. Also, nanoparticles prepared by combining chitosan, tripolyphosphate and chondroitin sulfate could be used to slowly release osteogenic proteins, such as Nel-like molecule-1 [79], in future bone graft applications.

An important bioactive molecule in guided bone regeneration is hydroxyapatite, and consequently, several composite hydroxyapatite-containing nanomaterials have been developed: composite chitosan/hydroxyapatite nanofibers [15,80,81], chitin hydrogel/ nanohydroxyapatite nanocomposite scaffolds [82], chitosan/polyvinyl alcohol/nanohydroxyapatite nanoscaffolds [83], and hydroxyapatite/ collagen/ chitosan nanocomposite fibers [84]. Further, biocomposite nanofibrous scaffolds have been fabricated from poly(3-hydroxybutyrate-co-3-hydroxyvalerate), chitosan and hydroxyapatite for mineral deposition [85]. In the case of the latter, chitosan provided cell recognition sites, while hydroxyapatite acted as a chelating agent for organizing the apatite-like mineralization [85]. Interestingly, biocompatible coatings of cellulose acetate nanofibers and composites of hydroxyapatite nanoparticles and chitosan led to the formation of a bone-like apatite layer on the implants with such coverings [86]. One study found that hydroxyapatite mineralized on chitosan-coated poly(lactic acid) nanofiber composites could mimic structural, compositional, and biological functions of native bone [87].

**Table 2:** Main applications of composite chitosan nanosystems in the oral cavity.

Chitosan Composites	Dental Field	Year	Study
Chitosan-gold nanoparticles	implantology	2015	[70]
chitosan-poly(ε-caprolactone) nanofibers	bone regeneration	2015	[76]
chitosan, tripolyphosphate and chondroitin sulfatenanoparticles	bone regeneration	2012	[75]
composite chitosan/hydroxyapatite nanofibers	bone regeneration	2008	[80]
chitin hydrogel/nanohydroxyapatitenanocomposite scaffold	bone regeneration	2011	[82]
chitosan/polyvinyl alcohol/nanohydroxyapatitenanoscaffolds	bone regeneration	2008	[83]
hydroxyapatite/collagen/chitosan nanocomposite fibers	bone regeneration	2010	[84]
poly-3-hydroxybutyrate-co-3-hydroxyvalerate/chitosan/hydroxyapatite nanofibrous scaffold	bone regeneration	2015	[85]
chitosan/polyethylene oxide nanofibers	cartilage tissue engineering	2005	[88]
chitosan/polyvinyl alcohol nanofibers	oral candidiasis	2015	[89]
poly(ε-caprolactone)-poly(ethylene glycol) copolymernanofibrous mats incorporated into chitosan	regeneration of periodontium	2015	[90]
chitosan/polyethylene oxide nanofibers	wound healing	2014	[91]
poly(ε-caprolactone)/chitosan nanofibers	wound healing	2014	[92]
chitosan/polyvinyl alcohol nanofibers	wound healing	2015	[93]
chitosan/collagen nanofibrous membranes	wound healing	2006	[94]
chitosan hydrogel/nanofibrin composite	wound healing	2012	[95]
chitosan/sericin nanofibers		2014	[96]
chitosan-Eudragit nanofibrous sheets		2015	[97]
chitosan/arginine nanofibrous membrane		2015	[98]
chitosan/gelatin/shape memory polyurethane nanofibers		2015	[99]
tannic acid/chitosan/pullulan composite nanofibers		2015	[100]
chitosan-rose bengal nanoparticles		2014	[101]
			

There is also a high number of research papers that have developed composite chitosan-containing nanofibers for other biomedical applications: nanofibers of chitosan/polyethylene oxide for cartilage tissue engineering [88], chitosan/polyvinyl alcohol nanofibers for oral candidiasis treatment [89,102] and poly(ε-caprolactone)-poly(ethylene glycol) copolymer nanofibrous mats that were incorporated into chitosan for the regeneration of periodontium [90]. An interesting multitude of nanocomposite materials have been fabricated for wound healing applications: chitosan/polyethylene oxide nanofibers [91,103], poly(ε-caprolactone)/chitosan nanofibers [92,104,105], chitosan/polyvinyl alcohol nanofibers [93,106,107], chitosan/collagen nanofibrous membranes [94], chitosan hydrogel/nanofibrin composite [95], chitosan/sericin-nanofibers [96], chitosan-Eudragit nanofibrous sheets [97], chitosan/arginine nanofibrous membrane [98], chitosan/gelatin/shape memory polyurethane nanofibers [99] or tannic acid/chitosan/pullulan composite nanofibers [100].

Different studies reported on using CNP antibiotic qualities in odontotherapy [40,108], but this functionality is questionable, as some authors have stated that the pulpal tissue has a strong inhibitory effect on CNP antibacterial characteristics. Composite chitosan nanoparticles have been fabricated because of their enhanced antibacterial property, which is important in anti-cavities therapy or endodontic procedures. One study developed a nanosized compound which contains silver nanoparticles, chitosan and fluoride, which seems to be a promising anti-cavities agent with low toxicity to living cells [109]. Several chitosan nanoparticles, nanofilms or nanofibers have been also incorporated with silver in order to enhance the antibacterial activity of the nanocomposites [110,111,112,113]. Researchers developed photoactivated Rose Bengal-functionalized chitosan nanocarriers that were effective in killing *Enterococcu sfaecalis* biofilms [101,114,115], as well as in neutralizing lipopolysaccharides obtained from *Pseudomonas aeruginosa* while also stabilizing dentin-collagen. Taking into account that *E. faecalis* and *P. aeruginosa* are associated with endodontic infections [116], their killing would be beneficial in dental endodontic treatments.

This multitude of available research papers supports the idea that chitosan nanoparticles are one of the most studied organic nanosystems in the dental field. Nanocomposite CNPs development is significant because of their improved properties, mostly in guided bone regeneration procedures or in dental therapies where the ability to accurately kill bacteria is necessary. Following this trend, it is expected that the development of new composite chitosan nanoparticles will rise even further in the following years.

### 2.4. Silk Fibroin Nanoparticles

Silk fibroin nanoparticles have been used in a multitude of medical applications (Table 3), mainly in regenerative medicine, because of their controlled delivery properties [117]. Silk fibroin can be modified by adding different functional groups. Functionalization allows for an adjustable drug release system with distinct interactions between drug and nanocarrier and varied releasing kinetics. The kinetics are also dependent on silk fibroin’s large molecular weight, hydrophobicity and crystalline structure. Details on the chemistry, structure and delivery properties of silk fibroin can be found elsewhere [118]. Nanosystems made from silk fibroin showed encouraging results in studies addressing regeneration of tissues found in the oro-maxillo-facial field, such as bone, skin or vascular tissues. Although not the main focus of regenerative dentistry, wound healing and vascular tissue formation are also important for the dentist, considering the frequency of oral mucosa wounds found in everyday practice. Silk fibroin nanoparticles could have numerous applications in dentistry, but mainly in bone regeneration procedures, where they have been used in the form of nanospheres, nanofibrous membranes, or nanofibrous scaffolds [117]. New bone formation is needed in dental surgeries such as implant therapy or in some periodontal treatments. Studies found that experimental nanofibrous electrospun silk scaffolds incorporated with bone morphogenetic protein-2 supported human mesenchymal stem cells osteogenic differentiation [119]. Silk fibroin nanofiber membranes were implanted in calvarial defects of rabbits and resulted in complete healing with new bone after 12 weeks [120]. Also, silk fibroin nanospheres were applied in the manufacture process of vaterite-microparticles, which could be further used as drug carriers in bone tissue engineering [121]. Moreover, composite nanostructures of silk fibroin and chitosan were developed, combining the advantages of both organic materials. Nanofibrous membrane scaffolds of chitosan and silk fibroin led to osteogenic differentiation of human mesenchymal stem cells, as was demonstrated by the increased alkaline phosphatase activity and expression of the osteogenic marker genes [122]. In a recent animal study, nanohydroxyapatite was added to the chitosan/silk fibroin nanofibrous membrane, thereby facilitating the osteogenesis of human mesenchymal stem cells [123]. Also recently, a thin nanofibrous membrane containing silk fibroin/chitosan/ nanohydroxyapatite/ bone morphogenetic protein-2,subcutaneously implanted together with human mesenchymal stem cells, resulted in ectopic bone formation *in vivo* [124]. Wound healing in the oral cavity would suggest a wound dressing material which is possible to contain silk fibroin nanosystems. Hybrid or blend chitin/silk fibroin nanofibers could be effective as wound-healing accelerators in tissue regeneration [125]. The chitin/silk fibroin blend nanofibrous matrix, containing 75% chitin and 25% SF, has been shown to promote excellent cell attachment and spreading for human keratinocytes and fibroblasts [126]. It is important to note that pure silk fibroin nanofibers led to enhanced spreading of human epidermal keratinocytes than the pure chitin nanofibers. In order to improve the antibacterial proprieties of the wound dressing materials, one experiment developed nanofibrous membranes of chitosan and silk fibroin effective against *E. coli*, a gram negative bacteria [127]. The potential of silk fibroin nanofibers to form vascular tissues should also be noted. Nanofibers of silk fibroin and chitosan managed to support human umbilical vein endothelial cells into forming capillary-like vascular structures and could be used to regenerate vascular tissue [128]. Although there are no specific studies on silk fibroin nanoparticles use in oral cancer, silk fibroin nanoparticles can deliver drugs and genes to the tumorigenic cells [129,130]. Nanocarriers of silk fibroin loaded with the antitumor drug cisplatin were able to efficiently deliver the drug to cancer cells while avoiding the cytotoxicity and side effects of the free drug on normal tissues [130]. Considering all this, it may be possible to speculate that silk fibroin nanoparticles could have future applications in the field of oral cancer treatment. While *in vitro* experimental procedures have shown very promising results, future studies are needed for assessing silk fibroin nanoparticles properties in animal studies. Silk fibroin is an attractive biomaterial for tissue engineering and controlled delivery of molecules but as in the case of chitosan several challenges remain. As natural products their characteristics may vary between individuals and species. To date, in the dentistry, the main focus of silk fibroin drug delivery systems has been on bone regeneration applications and wound healing products.

**Table 3:** Applications of silk fibroin nanoparticles in dentistry.

Nanoparticle Type	Dental Field	Type of Study	Study
silk fibroin nanofiber membranes	bone tissue engineering	*in vivo*	[120]
silk fibroin nanofibrouselectrospun scaffolds	bone tissue engineering	*in vitro*	[119]
silk fibroin/chitosan nanofibers	bone tissue engineering	*in vitro*	[122]
silk fibroin/chitosan/nanohydroxyapatite nanofibrous membrane scaffolds	bone tissue engineering	*in vivo*	[123]
silk fibroin/chitosan/nanohydroxyapatite nanofibrous membrane	bone tissue engineering	*in vivo*	[124]
silk fibroin/chitin blend fibers	wound healing accelerator	*in vitro*	[125]
silk fibroin and chitosan nanofibrous membranes	wound healing accelerator	*in vitro*	[127]
silk fibroin and chitosan nanofibers	vascular tissue regeneration	*in vitro*	[128]

## 3. Synthetic Organic Nano-Delivery Systems

Full exploitation of the natural materials has been limited because of the batch-to-batch variations in their properties [131]. On the other hand, manufacturing of synthetic polymers has the flexibility and reproducibility desired for nanomedical applications [132]. In dental medicine, one of the most studied synthetic organic molecule is poly-lactic-co-glycolic acid co-polymer. PLGA is highly compatible and has been approved by the U.S. Food and Drug Administration for the use of drug delivery, diagnostics and other medical applications. Most importantly, PLGA is biodegradable; its degradation leads to carbon dioxide and water [133].

### 3.1. PLGA Nanoparticles

The number of studies published on PLGA nanoparticles (NPs) application in dentistry has grown in recent years [8]. Recently, a wide variety of studies has been undertaken leading the way for possible future applications of PLGA NPs in a high number of dental fields, from periodontology [134] and endodontics [135] to tissue regeneration of skin [136,137,138], bone [139], or cartilage [140,141,142,143,144] (Table 4).

**Table 4:** Applications of PLGA nanoparticles in the dental field.

Active Substance	Dental Field	Year	Study
photosensitizer methylene blue	endodontics	2010	[135]
antibiotic minocycline	periodontics	2012	[134]
parathyroid hormone	bone regeneration	2015	[139]
recombinant human bone morphogenetic protein-7	bone regeneration	2007	[145]
nafcillin	osteomyelitis treatment	2008	[146]
simvastatin	osteoporosis treatment	2015	[147]
lovastatin	healing of fractures	2007	[148]
LL37 (a human host defense peptide)	wound healing	2014	[137]
curcumin	wound healing	2013	[136]
vascular endothelial growth factor	wound healing	2015	[138]
dexamethasone	gingival fibroblast differentiation	2015	[149]
amphotericin B	fungal infections treatment	2015	[150]
chondrogenesis related proteins	chondrogenesis	2014	[142]
Genes SOX 5, SOX 6, SOX 9	chondrogenesis	2011	[140]

The most important studies that focus on PLGA NPs and their uses in the dental field are described below. PLGA nanoparticles loaded with the photosensitizer methylene blue exhibited significant killing of *Enterococcus faecalis* biofilm species in experimentally infected root canals of extracted human teeth [135]. These results are promising, considering that *Enterococcus faecalis* is highly associated with endodontic treatment failure [151,152]. Moreover, encapsulation of the antibiotic mynocycline into PLGA nanoparticles proved to be remarkably more effective than the free drug against *Aggregatibacter actinomycetemcomitans* [134], another etiologic agent of periodontal diseases [153,154,155]. Another research direction with potential clinical applications is the use of PLGA to support new bone formation [156,157,158,159,160,161] and/or osteogenic differentiation [162]. PLGA NPs encapsulated with recombinant human bone morphogenetic protein-7 in a nanoporouspoly(l-lactic acid) scaffold induced bone healing in rats [145]. Additionally, nanocarriers consisting of PLGA and incorporating bioactive molecules nafcillin [146] or simvastatin [147] were studied for osteomyelitis and osteoporosis treatment, respectively .PLGA NPs could also be utilized in fracture treatment, as one study found that lovastatin-containing PLGA nanosystems increased the rate of healing of femoral fractures [148]. Interestingly, nanosized PLGA had positive results in experiments concerning wound healing applications [136,137,138]. LL37(a human host defense peptide) encapsulated in PLGA nanoparticles led to nearly complete wound closure in mice by day 13 due to the sustained release of both LL37 and lactate. Similarly, curcumin and vascular endothelial growth factor in PLGA nanostructures promoted re-epithelization or healing of non-diabetic and diabetic wounds. Cartilage regeneration might be a promising novel approach for treating articular fractures or disorders of the temporo-mandibular joint. Numerous experiments obtained chondrogenesis of human mesenchymal stem cells by exposing cells to PLGA NPs that incorporated chondrogenesis-related proteins, transcription factors, or genes. Of interest also is the fact that PLGA nanocarriers have been developed to deliver active molecules for other possible dental-related applications: transport of dexamethasone for gingival fibroblasts differentiation [149] or controlled release of amphotericin B for fighting against fungal infections [150]. Novel skin cancer treatments could include PLGA NPs [163] transporting small molecules, RNA or genes in order to target the p53 inactivation or the epidermal growth factor receptor over-expression in skin squamous carcinoma tumors [164].Taking into account the numerous experiments involving PLGA and PLGA-derived nanoparticles used for diagnosis or treatment of other types of tumors [13,165,166,167,168], an increase in research regarding PLGA nanoparticles uses in the oral cancer field is expected.

### 3.2. Composite PLGA Nanoparticles

PLGA materials are also easy to fabricate and combine with a wide variety of natural or synthetic molecules in different shapes and structures (nanofibers, nanocontainers or nanoscaffolds). Interestingly, PLGA and chitosan are some of the most studied organic polymers in dentistry. Because of chitosan’s biocompatibility, the PLGA-chitosan combination in biomedical nanostructures creates promising applications in prophylactic dentistry [30] or wound healing [169,170]. Chitosan and chitosan-covered PLGA nanoparticles could be integrated in dental toothpaste, as indicated by one study where nanoparticles were loaded with chlorhexidinedihydrochloride [30]. The release of the active molecules from chitosan NPs was dependent on the pH of the medium, while PLGA nanocarriers’ release of chlorhexidine was less pH-dependent [30]. Moreover, PLGA/chitosan nanofibers promote the fibroblasts’ attachment and proliferation and could therefore be used in skin tissue engineering, while their functionalization with graphene oxide and silver nanoparticles creates a biomaterial with antimicrobial proprieties against both Gram-negative (*Escherichia coli* and *Pseudomonas aeruginosa*) and Gram-positive (*Staphylococcus aureus*) bacteria [171].

Although PLGA nanofibers are widely used in the manufacture of scaffolds for tissue regeneration [171] and despite their biocompatibility, the clinical applications of pure PLGA for bone regeneration are hampered by its poor osteoconductivity [172,173]. Therefore, a variety of composite PLGA scaffolds have been developed for bone tissue engineering (Table 5). Different nanosized PLGA composite systems have been successful at inducing osteogenic differentiation of stem cells: PLGA/collagen nanofibers with calcium phosphate [174], PLGA/ poly(3-hydroxybutyrate-co-3-hydroxyvalerate) nanoparticles [175], nanoparticles of bis(poly(lactic-co-glycolic acid)-phenylalanine-polyethylene glycol-quaternary ammonium grafted diethyltriaminbis (PLGA-phe-PEG)-qDETA) [176], and even PLGA-hyaluronic acid copolymer nanoparticles [177]. Some of these composite nanostructures, such as PLGA/collagen/calcium phosphate nanomembrane [174], managed to support osteogenic differentiation on their own, while others were incorporated with simvastatin [176] or growth factors like bone morphogenetic protein-2 [178], BMP-2 and BMP-7 [45], bone morphogenetic protein-2 and insulin-like growth factor-1 [177]. Interestingly, although the mechanism is not fully understood, the bis(PLGA-phe-PEG)-qDETA nanoparticles alone were able to promote the osteogenesis of the bone marrow mesenchymal stem cells, possibly by enhancing the expression of osteocalcin that leads to elevated alkaline phosphatase (ALP) expression and mineralization [177].

**Table 5:** Main applications of composite PLGA nanosystems in dental medicine.

PLGA Composite Nanosytems	Dental Field	Year	Study
PLGA nanoparticles covered with chitosan	dental toothpastes	2015	[30]
PLGA/chitosan nanofibers	wound regeneration	2014	[170]
Calcium phosphate/collagen/PLGA nanofibers	bone regeneration	2011	[174]
PLGA/poly(3-hydroxybutyrate-co-3-hydroxyvalerate) nanoparticles	bone regeneration	2010	[175]
bis(poly(lactic-co-glycolic acid)-phenylalanine-polyethylene glycol-quaternary ammonium grafteddiethyltriamin nanoparticles	bone regeneration	2014	[176]
PLGA-HA copolymer nanoparticles	bone regeneration	2014	[177]
PLGA/polycaprolactonenanoparticles	bone regeneration	2015	[178]
heparin-fibrin-poly(lactide-co-caprolactone) nanoparticles	chondrogenic differentiation	2009	[179]
PLGA nanoparticlescovered with hyaluronic acid	osteogenic differentiation	2015	[180]

There is also one in vivo study that used polycaprolactone/PLGA injectable nanoparticles containing recombinant human BMP-2 in rabbits [178]. The results were very positive, with 78% trabecular bone formation with surrounding fibro-vascular tissues within 6 weeks. Future treatments of joints or articular structures may be based on cartilage regeneration. Heparin-fibrin-poly(lactide-co-caprolactone) nanoparticle complex releasing transforming growth factor beta 1 sustained chondrogenic differentiation of human adipose derived stem cells [179], while hyaluronic acid-covered PLGA nanoparticlescould have future applications in chondrogenic, osteogenic or adipogenic new tissue formation [180].

### 3.3. Dendrimers, Lipid Nanoparticles and Liposome Applications in the Dental Field

Dendrimers are highly branched, synthetic polymers with micelle-like behavior demonstrating promising results in several biomedical applications. Their properties make them suitable as scaffolds for tissue repair, targeted carriers for antiviral or chemotherapeutic drugs, gene delivery systems, and as ligands for various medical applications [181,182]. Dental applications of dendrimers are currently limited and research in the field demonstrates conflicting results depending on the dental field in which the particle have been used and on the polymer choice. Reports show that dendrimers may be used as releasing-scaffolds for releasing anti-periodontopathogenic agents [183]; implant surface coating with phosphoserine and polylysine-dendrimers do not improve their osteointegration [184] while other studies report that they can promote osteoblast differentiation [185]; poly-amidoamine-dendrimer molecules have anti-adhesive properties and modulate the oral bacterial response [186]; dentine surface coating with poly-amidoamine-dendrimers can induce hydroxidapatite formation and thus contribute to dentinal tube occlusion [187,188,189].

Solid lipid nanoparticles are a valuable drug carrier systems, representing a solid alternative to carriers such as micro-/nanoparticles or liposomes. These particles have several attributes that make them attractive as drug delivery systems: physically stable system; small size (typically from 50 to 100 nm); high drug loading; large surface area when compared to their size; low toxicity, improved delivery of lipophilic active compounds. However their usage in the medical field is limited by several shortcomings such as: systems have a tendency to gelate over time resulting in expulsion of the drug and an increase in size over time [190]. There are very few studies that focus on the interaction between these nanoparticles and the biology of oral cavity. To date potential applications of lipid nanoparticles in the oral field such as in the treatment of periodontal diseases, are lacking.

Liposomes are spherical vesicles with a diameter ranging from 20 nm to several micrometres, consisting of one or more lipid bilayers surrounding aqueous spaces [191]. They are made from natural lipid molecules, mainly phospholipids [191] and are considered to be non-toxic, non-immunogenic and biodegradable [192,193]. Liposomes are one of the most employed nanodevices to encapsulate antibiotics for treating intracellular infections [194] and therefore, could have promising applications in periodontology. Numerous research groups have fabricated antibiotic incorporating liposomes for use against various periodontal pathogens including liposomes containing metronidazole against *Streptococcus mutans* [195], chlorhexidine and triclosan for *Streptococcus oralis* [196], triclosan against *Streptococcus sanguis* [197] or for *Pseudomonasaeruginosa*, as well as amikacin and gentamicin-containing nanoliposomes [198].Similarly, the combination of liposomes and photodynamic therapy was proven effective against *Porphyromonasgingivalis* [199]. In addition liposomes incorporating bovine lactoferrin (LbLF)could be used for periodotal prevention in patients undergoing orthodontic treatments [200]. Recent *in vivo* studies suggested that liposomes significantly inhibited lypopolysacharide induced bone resorption but not orthodontic force induced bone remodeling. Similarly, scaling and root planing with subgingival application of liposome-encapsulated SOD managed to suppress periodontal inflammation on experimentally induced periodontitis in beagle dogs, after only 6 weeks of treatment [200]. Moreover, liposomal phosphatidylserine inhibits osteoclastogenesis and adjuvant arthritis-induced trabecular bone loss in rats, by generating TGF-β1 and PGE_2_ [201].Another characteristic with implications in the dental field is the ability of liposomes to target macrophages naturally. Thus mynocycline hydrochloride nanoliposomes could provide a long term therapeutic effect in targeted, controlled release topical periodontitis therapy, they could inhibit the proliferation of murine macrophages (ANA-1), and specifically achieve anti-inflammatory effects by suppression of TNF-α mRNA expression. Furthermore, liposomes were able to deliver plasmid DNA into the gingiva and could have future applications in periodontal genetic therapies [202]. However, these nanostructures are not considered very stable, especially the larger size liposomes [203] and that is a significant drawback of liposome use in dental applications.

Among all nanomaterials presented in this review, some have the potential of being translated to clinical situations more than others. For example, one promising application for PLGA nanoparticles that could develop into a commercial application is described by Sadat *et al.* [134]. The PLGA nanoparticles are reported to release minocycline over a 5-day period, suggesting that this nanosystem can be ideal for treatment of periodontal diseases. Similarly, the use of PLGA nanoparticles loaded with the photosensitizer methylene blue showed bactericidal effects on biofilm species in infected root canals of human teeth and may have promising results in antimicrobial endodotic treatment [135]. Both chitosan and silk fibroin based nanoparticles have been shown to efficiently encapsulate and deliver antitumor drugs. However the reviewed literature shows that in dentistry these two polymers are more suitable for applications focusing on tissue engineering (especially bone formation), formation of vascular tissue and wound healing (Table 2 and Table 3).

## 4. Conclusions

In the light of the above studies, the application of nanotechnology-based dentistry stands to profit greatly from the development of organic-based materials. A variety of shapes and structures, from nanomembranes, nanopowders and nanofibers to nanoscaffolds or nanogels can be fabricated from organic materials. While liposomes, solid lipid and dendrimers have been developed to address oral disease treatment, polymeric nanoparticles made of PLGA, chitosan and silk fibroin appear to be some of the most studied nanomaterials in dentistry.

Nanostructures fabricated from these materials support regeneration of oral tissues, covering all main dental fields from periodontology and endodontics to bone healing. The available literature shows that, to date, PLGA is the most used polymer for NPs in dentistry. Its properties make it especially suitable as a reliable drug delivery system, but so far there is no general consensus on the benefits of PLGA nanoparticles when used alone in bone regeneration therapies. Moreover, surface coating with these polymeric NPs can have little or no effect on the dental implant osteintegration. One concern is related to the fact that several oral bacteria have good adherence on PLGA which can potentially lead to infections for *in vivo* applications. Chitosan and silk fibroin are more often selected for oral tissue engineering. Chitosan is recognized for its mucoadhesivity and the property of improving active compounds’ penetration across mucosal surfaces, while also supporting cellular adhesion, mobility and proliferation. In an increasing trend, composite nanostructures are being developed, combining advantages of all individual incorporated materials. Chitosan nanoparticles are easily combined with other substances, conferring or improving antimicrobial and antifungal proprieties to a variety of nanostructures. Additionally, silk fibroin nanoparticles, due to their controlled delivery properties, exhibit promising results in studies for formation of oral bone or vascular tissue.

Several limitations of polymeric nanoparticle use in dentistry are noted. PLGA is a co-polymer that can be degraded by hydrolysis to the two initial acids and, unlike chitosan or silk fibroin, can alter the pH at the delivery site, affecting the surrounding tissue as well as the effectiveness of the delivered drug. Other pitfalls related to PLGA-based NPs, include their relative poor loading of the active compound coupled with a high release burst of the drug. Overall, poor drug loading efficiency seems to be the major issue for PLGA NPs and can limit the use of these particles in clinical trials. Biomacromolecules’ instability inside polymeric particles is another important issue to consider. It has been reported that the structure of fragile molecules such as DNA, RNA or proteins can be destabilized or degraded during drug entrapment. A significant limitation of natural components chitosan and silk fibroin is variations in their properties as a result of vendor-dependent extraction and purification methods. Another limitation of organic NPs in general, is the relatively insufficient data on their interaction and impact on the oral environment. For all types of nanoparticles described in this review more data is needed in order to fully understand the interaction of these nanocarriers with cells and tissue and to assess their toxic potential. While most *in vitro* and animal model studies report encouraging results, clinical trials are needed in order to correctly assess NPs toxicity *in vivo*.

The field of organic composites used for controlled delivery of antibiotics, growth factors or even anticancer substances continues to grow. Based on the positive data obtained *in vivo* and *in vitro*, further studies are needed in order to translate the knowledge obtained in the research lab to the daily clinical dental practice.
